# Promising Applications of Nanoparticles in the Treatment of Hearing Loss

**DOI:** 10.3389/fcell.2021.750185

**Published:** 2021-10-07

**Authors:** Zilin Huang, Qiang Xie, Shuang Li, Yuhao Zhou, Zuhong He, Kun Lin, Minlan Yang, Peng Song, Xiong Chen

**Affiliations:** ^1^Department of Otorhinolaryngology, Head and Neck Surgery, Zhongnan Hospital of Wuhan University, Wuhan, China; ^2^Sleep Medicine Center, Zhongnan Hospital of Wuhan University, Wuhan, China

**Keywords:** hearing loss, nanomaterials, drug delivery system, imaging, hair cells, cochlear implant

## Abstract

Hearing loss is one of the most common disabilities affecting both children and adults worldwide. However, traditional treatment of hearing loss has some limitations, particularly in terms of drug delivery system as well as diagnosis of ear imaging. The blood–labyrinth barrier (BLB), the barrier between the vasculature and fluids of the inner ear, restricts entry of most blood-borne compounds into inner ear tissues. Nanoparticles (NPs) have been demonstrated to have high biocompatibility, good degradation, and simple synthesis in the process of diagnosis and treatment, which are promising for medical applications in hearing loss. Although previous studies have shown that NPs have promising applications in the field of inner ear diseases, there is still a gap between biological research and clinical application. In this paper, we aim to summarize developments and challenges of NPs in diagnostics and treatment of hearing loss in recent years. This review may be useful to raise otology researchers’ awareness of effect of NPs on hearing diagnosis and treatment.

## Introduction

Hearing loss is one of the most common disabilities affecting the quality of life. Nowadays, people’s lifestyle has been changed with longer life expectancy, and the prevalence and the severity of hearing loss have increased (Cruickshanks et al., 2003; Isaacson, 2010). According to World Health Organization (WHO), more than 5% of the world’s population suffer from disabling hearing loss that includes 34 million children (Chadha and Cieza, 2017), and it is more prevalent in the elderly (≥70 years) (Zahnert, 2011). Hearing loss is divided into three categories: conductive, sensitive, and mixed hearing loss. Common causes of conductive hearing loss are earwax embolism, otitis media, cholesteatoma, and otosclerosis, among others (Zahnert, 2011). Sensorineural hearing loss (SNHL) is usually caused by sensory nerve transmission problems at or behind the cochlea, including presbycusis, inner ear infection (He et al., 2020), Meniere’s disease (Wang et al., 2015), noise-induced hearing loss (Varela-Nieto et al., 2020), autoimmune hearing loss (Fan et al., 2019), genetic diseases (Zhu et al., 2018; Cheng et al., 2021; Fu et al., 2021a; Lv et al., 2021), age-related hearing loss (He et al., 2021), and ototoxic material hearing loss (Liu et al., 2016, 2021; Gao et al., 2019; Liu W. et al., 2019; Zhang Y. et al., 2019; Zhong et al., 2020; Fu et al., 2021b).

Attention to the treatment of hearing loss has varied, which is influenced by social status, education, and race. For example, nearly two-thirds of United States adults aged 70 years and older are affected by hearing loss, and only 15% of older people use hearing aids (Mamo et al., 2016). At present, the traditional treatment of hearing loss includes drug therapy, hearing aids, and cochlear implant (CI). Systemic administration and intratympanic (IT) steroid injection are much prevalent clinical therapy to restore hearing loss (Ermutlu et al., 2017; Mirian and Ovesen, 2020). Due to the special and complex anatomical structure of the inner ear, the blood–labyrinth barrier (BLB) prevents most drugs in the blood from reaching the inner ear, such as protein, carbohydrate, and other small molecules (Shi, 2016); most of the hearing loss drug treatment is ineffective (Nyberg et al., 2019). Compared to systemic administration, IT injection has been shown to keep high concentrations of steroids in the perilymph and can be used as a substitute or supplement for systemic steroid therapy (Chandrasekhar et al., 2000). However, there are also differences in round window membrane (RWM) size and permeability in IT injection, which makes it difficult to accurately determine the drug concentration for individualized treatment (Goycoolea, 2001).

Hair cells are the mechanical transduction cells in the cochlea, which detect sound through the deflection of mechanosensory stereocilia, and are the most critical cells in the inner ear (He et al., 2017, 2019; Liu Y. et al., 2019; Qi et al., 2019, 2020; Zhou et al., 2020). Once damaged, hair cells only have very limited regeneration ability in mammals, and it is difficult for the new neuron cell to proliferate in a specific site (Cheng et al., 2019; Tan et al., 2019; Zhang S. et al., 2019; Zhang et al., 2020b; Chen et al., 2021). Possibly, hair cells are so fragile that the generation of inflammation in the inner ear can affect hair cell survival, and the protection of these cells is the key to the treatment of hearing loss (Zhang et al., 2020a,c). Because of the limitations of traditional treatment of hearing loss, nanomaterials are more and more likely to appear in the treatment of inner ear diseases as a new type of small medical molecular particles (Liu et al., 2018; Han et al., 2020; Yuan et al., 2021; Zhao et al., 2021). Nanoparticles (NPs) with a diameter of 1–1,000 nm can not only promote the effective concentration time of drugs *in vivo*, but also carry drugs to specific parts of the cochlea (Pyykkö et al., 2016). NPs have been demonstrated to have high biocompatibility, good degradation, and simple synthesis in the process of diagnosis and treatment (Zha et al., 2016; Shang et al., 2018; Yang et al., 2018; Zhao et al., 2019). Because of this, nanomaterials and their related products have been widely used in drug delivery applications, including cancer treatment, diagnosis, molecular imaging, and other applications (Shaikh et al., 2018; Li D. et al., 2019; Guo J. et al., 2020; Yang et al., 2021). Also, it is possible for nanomaterials to be used in hearing loss with many advantages that have been found in many other diseases’ treatment, such as the regeneration of neural stem cells, the induced differentiation of neurons, and the transmission of some specific active substances in inner ear cells (He et al., 2016; Jiang et al., 2020; Xia et al., 2020; Yang et al., 2020). There are various kinds of medical nanomaterials for hearing loss, such as poly(lactic-co-glycolic-acid) NPs, silica NPs, magnetic NPs, and lipid NPs (Pyykkö et al., 2016). This review aims to summarize the useful nanomaterials emerging in the diagnosis and treatment of hearing loss in recent years.

## Comparison of Traditional Medicine Treatment and Nanomedicine in Hearing Loss

**FIGURE 1:**
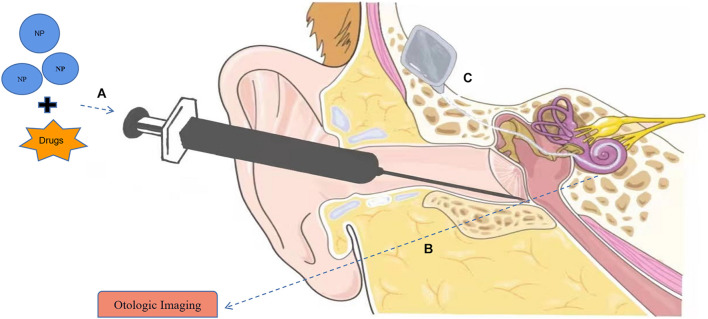
Applications of nanomaterials in hearing loss. **(A)** Nanoparticles (NPs) can serve in drug delivery systems to the inner ear with intratympanic (IT) injection. **(B)** Nanomaterials can be used as contrast agents in otologic imaging. **(C)** Application of nanomaterial in cochlear implants (CIs). Part of the material in Figure is from https://smart.servier.com.

At present, systemic drug delivery and IT injection (Figure 1) are the traditional drug treatments for hearing loss caused by inner ear diseases (Li et al., 2018). Previous studies have reported that systemic administration has been successfully used in the treatment of sudden hearing loss (SHL), autoimmune inner ear disease (AIED), Meniere’s disease, and other inner ear diseases by intravenous, intramuscular, or oral administration (Li and Ding, 2020; Liu et al., 2020). Although the drugs can reach the inner ear through systemic administration, the limited local blood supply and poor penetration of BLB often lead to the local drug concentration lower than the treatment criteria (Nyberg et al., 2019). In order to reach the expected therapeutic effect, large doses of drugs are needed, which often lead to serious ototoxicity. However, high dose of systemic glucocorticoids can lead to hypertension, hyperglycemia, osteoporosis, and immunosuppression, as well as long-term high-dose adrenal suppression (McCall et al., 2010; Stout et al., 2019), which is harmful to human health.

On the other hand, IT injects the drug into the middle ear space, allowing the drug to diffuse to the inner ear through the RWM, bypassing the labyrinthine artery and blood inner ear barrier, which is more efficient than systemic administration and avoids the side effects of high-dose medication (Buniel et al., 2009). Schuknecht (1957) first used IT injection as a means to deliver streptomycin into the inner ear to effectively treat the hearing loss of Meniere’s disease. The drug concentration of IT injection in the inner ear fluid, perilymph, and endolymph was significantly higher than that of oral or non-injection (Jackson and Silverstein, 2002; Buniel et al., 2009). Although IT administration is highly efficient and reduces the toxic and side effects of systemic administration, the concentration of drug reaching the inner ear depends on the dose of drug contacting the RWM circular window membrane of the middle ear, and the difference of RWM permeability will lead to the change of drug retention and elimination rate (Buniel et al., 2009; Lehner et al., 2021), which eventually makes it difficult to formulate a standard for dosing regimen. As a result, it is difficult to achieve precision therapy for hearing loss with traditional medication.

Compared with IT injection of dexamethasone, the products of nanotechnology have great advantages in drug treatment of hearing loss. For example, hydrogel nanomaterials deliver poloxamer 407 loaded with micronized dexamethasone (mDex) to guinea pig round window, which provides sustained release of drugs, increases the total concentration of peripheral blood lymphocytes by about 1.6-fold, and increases the residence time of drugs by about 24-fold. The initial peak concentration of dexamethasone injection before clearance from the lymphatic vessels was within 12 h, while the mDex hydrogel sustained release for 10 days (Wang et al., 2009). This study also demonstrates that mDex delivery using poloxamer 407 led to more homogenous distribution of dexamethasone along the length of the cochlea (Salt et al., 2011; Rathnam et al., 2019). In addition, some natural substances can also be transformed into NPs that promotes the growth of nerve cells or protect cells from inflammatory damage (Lambert et al., 2016), which means there will be more possibilities to find various natural NPs.

## Nanomaterials in Otology Imaging

As a new medical application of nanomaterials, the nano drug delivery system not only has a wide application in drug transportation of inner ear hearing loss, but also plays a role in clinical diagnosis and treatment of inner ear hearing loss diseases because of its specific penetration, good biocompatibility, and editability (Rathnam et al., 2019). Due to the special anatomical structure of BLB and the highly complex separation of the inner ear region, it is difficult to get enough contrast agents to reach the inner ear (Kayyali et al., 2017). Therefore, conventional Computed tomography (CT) and magnetic resonance imaging (MRI) are not appropriate for the imaging of the microstructure of the cochlea. However, it is difficult for many novel contrast agents with certain biocompatibility or targeting to guarantee the sensitivity and specificity of the inner ear diseases’ diagnosis (Liu et al., 2021).

Superparamagnetic nanoparticles (SPIONs) with good physical properties are characterized by nanocrystalline iron oxide (Fe_3_O_4_) or magnetite (γ-Fe_2_O_3_) nucleus, with a molecular diameter of 100–300 nm and with a certain biocompatibility (Laurent et al., 2008; Salazar-Alvarez et al., 2008). Therefore, many studies have verified its possibility as a new MRI contrast agent. Ceric ammonium nitrate oxidant stabilized γ-maghemite NPs could be detected in the inner ear using MRI after IT administration *in vivo* (Zou et al., 2017b). Besides, there are also some NPs such as superparamagnetic magnetohematite (γ-Fe2O_3_) NPs and lipid NPs that are designed to combine with traditional contrast agents to form chelates that can reflect the distribution of these contrast agents in the cochlea and form visual images (Zou et al., 2017a,b). Some metal ion NPs have great advantages in inner ear structure imaging. For example, the contrast enhancement rate of the new optical contrast agent containing nano silver clusters is more than 90%, and the ear veins can be detected much clearly (Chu et al., 2014; Ray et al., 2014). Interestingly, the nano chelate containing gold proved that the CT imaging effect of the inner ear structure was concentration gradient dependent in a certain range (Zou et al., 2015). These findings may indicate that they can be used as a potential nano template to visualize the cochlear structure in the middle ear granule in the future and to assess whether the drugs reach the designated site by positron emission tomography and MRI in the application of inner ear diagnosis and therapy.

## Application of Nanomaterials in Cochlear Implant

There are more than 324,000 CI users in the world. CI has greatly improved the quality of hearing life of patients with hearing loss. The mechanism of CI in the treatment of hearing loss is related to the connection between the CI electrode array and auditory neurons (Zhao et al., 2020). CI directly injects current into surrounding tissues through the implanted electrode array and maps the frequency of cochlea to location (Danti et al., 2020). Therefore, the emergence of nanotechnology makes significant innovation and progress of the CI electrode array. In order to reduce the damage caused by cochlear implantation, nanomaterials are applied to the corresponding electrode array to improve cochlear signal transmission and promote the growth of auditory nerve cells. Some physical stimulations as well as the influence of the cellular microenvironment are able to regulate cell migration and can direct neurite outgrowth in spiral ganglion neurons (SGNs) preferentially along a certain direction (Guo et al., 2019; Girão et al., 2020; Hu et al., 2021; Wei et al., 2021). More importantly, changes in cell culture environment can help to maintain and promote the electrophysiological properties of the SGNs, regulate the cells’ polarity, promote the area of growth cones, or significantly increase the synapse density of the SGNs (Sun et al., 2016; Yan et al., 2018).

The application of nanomaterials, such as graphene and MXene, can also promote the proliferation and differentiation of neural stem cells in the inner ear, and many ultrastructures can be produced by 3D printing technology or other novel methods, so as to obtain more satisfactory biological characteristics that can be applied in hair cells (Waqas et al., 2017; Fang et al., 2019; Xia et al., 2019; Guo R. et al., 2020; Guo et al., 2021). These new technological products indicate that the application of nanomaterials in cochlea may be conducive to hearing recovery and cell regeneration (Guo et al., 2016; Li G. et al., 2019; Tang et al., 2019). Similarly, biodegradable calcium phosphate hollow nanospheres, used as CI electrode coatings and loaded with neurothrophins, attract the growth of regenerating auditory neuron dendrites through bioactive gels and finally establish direct physical contact between the auditory neurons and the CI electrodes as a result (Li et al., 2017). Carbon nanotubes (CNTs) and micro-textured nano-crystalline diamond can also enhance the transmission of inner ear electrical stimulation by increasing the contact area of the coating, which brings no additional cell damage (Burblies et al., 2016; Cai et al., 2016; Choi et al., 2019).

What is more exciting is that there are also nanomaterials in the cochlea that can be used for a longer time by spontaneous power supply. Some studies have investigated the silver NP microcoil with the micro size by aerosol jet printing. It has been demonstrated that the electromagnetic field generated by this material is not affected by cochlear environment (Sarreal and Bhatti, 2020). The eddy current generated by electromagnetic field can be used to stimulate the nearby tissues, and to improve the spatial resolution of cochlear tissues and CI function (Golestanirad et al., 2018). Some researchers have also developed electrospun piezoelectric polymer nanofibers that can transform sound waves into electrical signals through the possible synergistic effect of piezoelectric and triboelectric, which provides a basis for the development of self-powered small nano cochlea (Viola et al., 2020).

These studies may have paved the way for the development of self-powered nanofibrous implantable auditory sensors, which suggests that more and more nanomaterials may be used in the construction of cochlear materials and cochlear signal transduction technology in the future.

## Discussion and Outlook of Nanomaterials in the Field of Hearing Loss

Nanomaterials and related technology products may not only provide diagnosis and treatment strategies for specific and efficient treatment of hearing loss, but also other inner ear diseases, such as otology tumors and ear inflammation. Furthermore, we can foresee that some types of nanomaterials or nanoproducts may be routinely used in the treatment of hearing loss and other inner ear diseases in the future (Li et al., 2017). Among the applications of nanomaterials in the diagnosis and treatment of hearing loss, researchers pay more attention to the biodegradability of nanomaterials and the ototoxicity *in vivo*. These substances act on the cells or tissues of the inner ear, which may also have ototoxicity, thus affecting the biological activity of hair cells and the activity of auditory neurons. Although some studies have found that NPs may have ototoxicity *in vivo*, there is no clear report on the ototoxicity of nanomaterials to humans (Murugadoss et al., 2021).

Previous studies have reported that positively charged NPs can enter the inner ear more easily through RWM, as drug carriers for inner ear diseases or CI materials, but they will produce certain ototoxicity in the process of biodegradation with cell membrane damage, production of reactive oxygen species, hair cell apoptosis, etc. (Yoon et al., 2015; Zhou et al., 2015). The time required for NPs to enter the body is longer than traditional drugs, but the possible effects of long-term residues of these NPs in animals are still unclear (Wang et al., 2009; Ray et al., 2014; Lehner et al., 2021). Therefore, future studies may need to determine whether the components of NPs will accumulate in the inner ear and the effects of these substances on hair cells.

Moreover, the cost of developing and manufacturing NPs for clinical application in the field of inner ear diseases is significantly higher than traditional treatments (Mokoena et al., 2019). Many patients with hearing loss may choose low-cost and convenient IT injection for treatment. Based on the current development of manufacturing technology, the manufacturing difficulty and cost of NPs are greatly overcome by printing technology, and it makes it easier for researchers to edit and manufacture NPs (Zhang et al., 2020d). In the future, we may choose to reduce the manufacturing cost of NPs through 3D printing, reduce the corresponding treatment costs, and try to produce NPs with more functions that are more convenient to be preserved or used. It is difficult to perfectly match the bioactivity of current nanomaterials to the conditions that are required for hair cell growth and proliferation in the inner ear, but it is possible that we may design nanomedicines that can precisely promote the differentiation of stem cells into auditory synesthesia cells, such as inner ear stem cells, mesenchymal stem cells, and pluripotent stem cells. In other words, if these novel nanomaterials can carry certain stem cells into the inner ear that promote stem cell differentiation into hair cells at specific structural locations, it will be a great advance in the treatment of hearing loss with significant hearing recovery.

## Conclusion

The application of nanomaterials in the diagnosis and treatment of hearing loss diseases is novel and promising. In the future, ideal nanomaterials should be more universal, able to load more therapeutic drugs with various functions, such as preventing rapid degradation, retaining targeting effects, and prolonging the action time in the inner ear. This kind of materials should not only have better efficacy in various diseases of inner ear hearing loss, but also have stronger ear permeability, and ensure no impact or side effects on the human body. Many studies have attempted to deliver drugs, genes, and growth factors to the inner ear *in vivo* with nanomaterials, and promising results have also been reported. However, we do not know the specific effect of nanomaterials applied in human inner ear. There is still a big gap between basic research and clinical application of nanomaterials, so it is necessary to study the safety and effectiveness of nanomaterials. With the emergence of new biomaterials and the realization of a deeper understanding of inner ear physiology, nanomaterials will have a clearer understanding of the diagnosis and treatment of hearing loss.

## Author Contributions

ZLH, SL, and QX wrote the first draft of the manuscript. YZ and ZHH polished the language. KL searched the literatures. XC, PS, and MLY conceived the idea and revised the manuscript. All authors read and approved the final manuscript.

## Conflict of Interest

The authors declare that the research was conducted in the absence of any commercial or financial relationships that could be construed as a potential conflict of interest.

## Publisher’s Note

All claims expressed in this article are solely those of the authors and do not necessarily represent those of their affiliated organizations, or those of the publisher, the editors and the reviewers. Any product that may be evaluated in this article, or claim that may be made by its manufacturer, is not guaranteed or endorsed by the publisher.

## References

[B1] Buniel M. C. Geelan-Hansen K. Weber P. C. Tuohy V. K. (2009). Immunosuppressive therapy for autoimmune inner ear disease. *Immunotherapy* 1 425–434. 10.2217/imt.09.12 19885385 PMC2747333

[B2] Burblies N. Schulze J. Schwarz H. C. Kranz K. Motz D. Vogt C. (2016). Coatings of different carbon nanotubes on platinum electrodes for neuronal devices: preparation, cytocompatibility and interaction with spiral ganglion cells. *PLoS One* 11:e0158571. 10.1371/journal.pone.0158571 27385031 PMC4934701

[B3] Cai Y. Edin F. Jin Z. Alexsson A. Gudjonsson O. Liu W. (2016). Strategy towards independent electrical stimulation from cochlear implants: guided auditory neuron growth on topographically modified nanocrystalline diamond. *Acta Biomater.* 31 211–220. 10.1016/j.actbio.2015.11.021 26593784

[B4] Chadha S. Cieza A. (2017). Promoting global action on hearing loss: world hearing day. *Internat. J. Audiol.* 56 145–147. 10.1080/14992027.2017.1291264 28262049

[B5] Chandrasekhar S. S. Rubinstein R. Y. Kwartler J. A. Gatz M. Connelly P. E. Huang E. (2000). Dexamethasone pharmacokinetics in the inner ear: comparison of route of administration and use of facilitating agents. *Otolaryngol. Head Neck Surg.* 122 521–528. 10.1067/mhn.2000.102578 10740171

[B6] Chen Y. Gu Y. Li Y. Li G. L. Chai R. Li W. (2021). Generation of mature and functional hair cells by co-expression of Gfi1, Pou4f3, and Atoh1 in the postnatal mouse cochlea. *Cell Rep.* 35:109016. 10.1016/j.celrep.2021.109016 33882317

[B7] Cheng C. Hou Y. Zhang Z. Wang Y. Lu L. Zhang L. (2021). Disruption of the autism-related gene Pak1 causes stereocilia disorganization, hair cell loss, and deafness in mice. *J. Genet. Genom.* 48 324–332. 10.1016/j.jgg.2021.03.010 34049799

[B8] Cheng C. Wang Y. Guo L. Lu X. Zhu W. Muhammad W. (2019). Age-related transcriptome changes in Sox2+ supporting cells in the mouse cochlea. *Stem Cell Res. Ther.* 10:365. 10.1186/s13287-019-1437-0 31791390 PMC6889721

[B9] Choi G. J. Gwon T. M. Kim D. H. Park J. Kim S. M. Oh S. H. (2019). CNT bundle-based thin intracochlear electrode array. *Biomed. Microdev.* 21:27. 10.1007/s10544-019-0384-y 30847585

[B10] Chu L. Wang S. Li K. Xi W. Zhao X. Qian J. (2014). Biocompatible near-infrared fluorescent nanoparticles for macro and microscopic in vivo functional bioimaging. *Biomed. Opt. Expr.* 5 4076–4088. 10.1364/boe.5.004076 25426331 PMC4242039

[B11] Cruickshanks K. J. Tweed T. S. Wiley T. L. Klein B. E. Klein R. Chappell R. (2003). The 5-year incidence and progression of hearing loss: the epidemiology of hearing loss study. *Archiv. Otolaryngol. Head Neck Surg.* 129 1041–1046. 10.1001/archotol.129.10.1041 14568784

[B12] Danti S. Azimi B. Candito M. Fusco A. Sorayani Bafqi M. S. Ricci C. (2020). Lithium niobate nanoparticles as biofunctional interface material for inner ear devices. *Biointerphases* 15:031004. 10.1116/6.0000067 32434336

[B13] Ermutlu G. Süslü N. Yılmaz T. Saraç S. (2017). Sudden hearing loss: an effectivity comparison of intratympanic and systemic steroid treatments. *Eur. Archiv. Otorhinolaryngol.* 274 3585–3591. 10.1007/s00405-017-4691-8 28756569

[B14] Fan K. Q. Li Y. Y. Wang H. L. Mao X. T. Guo J. X. Wang F. (2019). Stress-induced metabolic disorder in peripheral CD4(+) T cells leads to anxiety-like behavior. *Cell* 179 864–879.e19. 10.1016/j.cell.2019.10.001 31675497

[B15] Fang Q. Zhang Y. Chen X. Li H. Cheng L. Zhu W. (2019). Three-dimensional graphene enhances neural stem cell proliferation through metabolic regulation. *Front. Bioeng. Biotechnol.* 7:436. 10.3389/fbioe.2019.00436 31998703 PMC6961593

[B16] Fu X. An Y. Wang H. Li P. Lin J. Yuan J. (2021a). Deficiency of Klc2 induces low-frequency sensorineural hearing loss in C57BL/6 J mice and human. *Mol. Neurobiol.* 10.1007/s12035-021-02422-w [Epub ahead of print]. 34014435

[B17] Fu X. Wan P. Li P. Wang J. Guo S. Zhang Y. (2021b). Mechanism and prevention of ototoxicity induced by aminoglycosides. *Front. Cell. Neurosci.* 15:692762. 10.3389/fncel.2021.692762 34211374 PMC8239227

[B18] Gao S. Cheng C. Wang M. Jiang P. Zhang L. Wang Y. (2019). Blebbistatin inhibits neomycin-induced apoptosis in hair cell-like HEI-OC-1 cells and in cochlear hair cells. *Front. Cell. Neurosci.* 13:590. 10.3389/fncel.2019.00590 32116554 PMC7025583

[B19] Girão A. F. Sousa J. Domínguez-Bajo A. González-Mayorga A. Bdikin I. Pujades-Otero E. (2020). 3D reduced graphene oxide scaffolds with a combinatorial fibrous-porous architecture for neural tissue engineering. *ACS Appl. Mater. Interf.* 12 38962–38975. 10.1021/acsami.0c10599 32805917

[B20] Golestanirad L. Gale J. T. Manzoor N. F. Park H. J. Glait L. Haer F. (2018). Solenoidal micromagnetic stimulation enables activation of axons with specific orientation. *Front. Physiol.* 9:724. 10.3389/fphys.2018.00724 30140230 PMC6094965

[B21] Goycoolea M. V. (2001). Clinical aspects of round window membrane permeability under normal and pathological conditions. *Acta Otolaryngol.* 121 437–447. 10.1080/000164801300366552 11508501

[B22] Guo J. Yu Y. Sun L. Zhang Z. Zhao Y. Chai R. (2020). Bio-inspired multicomponent carbon nanotube microfibers from microfluidics for supercapacitor. *Chem. Eng. J.* 397:125517. 10.1016/j.cej.2020.125517 [Epub ahead of print].

[B23] Guo R. Li J. Chen C. Xiao M. Liao M. Hu Y. (2021). Biomimetic 3D bacterial cellulose-graphene foam hybrid scaffold regulates neural stem cell proliferation and differentiation. *Coll. Surf. B Biointerf.* 200:111590. 10.1016/j.colsurfb.2021.111590 33529926

[B24] Guo R. Ma X. Liao M. Liu Y. Hu Y. Qian X. (2019). Development and application of cochlear implant-based electric-acoustic stimulation of spiral ganglion neurons. *ACS Biomater. Sci. Eng.* 5 6735–6741. 10.1021/acsbiomaterials.9b01265 33423491

[B25] Guo R. Xiao M. Zhao W. Zhou S. Hu Y. Liao M. (2020). 2D Ti(3)C(2)T(x)MXene couples electrical stimulation to promote proliferation and neural differentiation of neural stem cells. *Acta Biomater.* 10.1016/j.actbio.2020.12.035 33348061

[B26] Guo R. Zhang S. Xiao M. Qian F. He Z. Li D. (2016). Accelerating bioelectric functional development of neural stem cells by graphene coupling: implications for neural interfacing with conductive materials. *Biomaterials* 106 193–204. 10.1016/j.biomaterials.2016.08.019 27566868

[B27] Han S. Xu Y. Sun J. Liu Y. Zhao Y. Tao W. (2020). Isolation and analysis of extracellular vesicles in a Morpho butterfly wing-integrated microvortex biochip. *Biosens. Bioelectron.* 154:112073. 10.1016/j.bios.2020.112073 32056968

[B28] He Z. H. Li M. Fang Q. J. Liao F. L. Zou S. Y. Wu X. (2021). FOXG1 promotes aging inner ear hair cell survival through activation of the autophagy pathway. *Autophagy* 17, 1–22. 10.1080/15548627.2021.1916194 34006186 PMC8726647

[B29] He Z. H. Zou S. Y. Li M. Liao F. L. Wu X. Sun H. Y. (2020). The nuclear transcription factor FoxG1 affects the sensitivity of mimetic aging hair cells to inflammation by regulating autophagy pathways. *Redox Biol.* 28:101364. 10.1016/j.redox.2019.101364 31731101 PMC6920089

[B30] He Z. Fang Q. Li H. Shao B. Zhang Y. Zhang Y. (2019). The role of FOXG1 in the postnatal development and survival of mouse cochlear hair cells. *Neuropharmacology* 144 43–57. 10.1016/j.neuropharm.2018.10.021 30336149

[B31] He Z. Guo L. Shu Y. Fang Q. Zhou H. Liu Y. (2017). Autophagy protects auditory hair cells against neomycin-induced damage. *Autophagy* 13 1884–1904. 10.1080/15548627.2017.1359449 28968134 PMC5788479

[B32] He Z. Zhang S. Song Q. Li W. Liu D. Li H. (2016). The structural development of primary cultured hippocampal neurons on a graphene substrate. *Colloids Surf. B Biointerf.* 146 442–451. 10.1016/j.colsurfb.2016.06.045 27395037

[B33] Hu Y. Li D. Wei H. Zhou S. Chen W. Yan X. (2021). Neurite extension and orientation of spiral ganglion neurons can be directed by superparamagnetic iron oxide nanoparticles in a magnetic field. *Intern. J. Nanomed.* 16 4515–4526. 10.2147/ijn.S313673 34239302 PMC8259836

[B34] Isaacson B. (2010). Hearing loss. *Med. Clin. North Am.* 94 973–988. 10.1016/j.mcna.2010.05.003 20736107

[B35] Jackson L. E. Silverstein H. (2002). Chemical perfusion of the inner ear. *Otolaryngol. Clin. North Am.* 35 639–653. 10.1016/s0030-6665(02)00023-3 12486845

[B36] Jiang P. Zhang S. Cheng C. Gao S. Tang M. Lu L. (2020). The roles of exosomes in visual and auditory systems. *Front. Bioeng. Biotechnol.* 8:525. 10.3389/fbioe.2020.00525 32582658 PMC7283584

[B37] Kayyali M. N. Brake L. Ramsey A. J. Wright A. C. O’Malley B. W. Li D. D. (2017). A novel nano-approach for targeted inner ear imaging. *J. Nanomed. Nanotechnol.* 8:456. 10.4172/2157-7439.1000456 29104815 PMC5669391

[B38] Lambert P. R. Carey J. Mikulec A. A. LeBel C. (2016). Intratympanic sustained-exposure dexamethasone thermosensitive gel for symptoms of ménière’s disease: randomized Phase 2b safety and efficacy trial. *Otol. Neurotol.* 37 1669–1676. 10.1097/mao.0000000000001227 27749754 PMC5414596

[B39] Laurent S. Forge D. Port M. Roch A. Robic C. Vander Elst L. (2008). Magnetic iron oxide nanoparticles: synthesis, stabilization, vectorization, physicochemical characterizations, and biological applications. *Chem. Rev.* 108 2064–2110. 10.1021/cr068445e 18543879

[B40] Lehner E. Liebau A. Syrowatka F. Knolle W. Plontke S. K. Mäder K. (2021). Novel biodegradable round window disks for inner ear delivery of dexamethasone. *Intern. J. Pharm.* 594:120180. 10.1016/j.ijpharm.2020.120180 33338566

[B41] Li A. You D. Li W. Cui Y. He Y. Li W. (2018). Novel compounds protect auditory hair cells against gentamycin-induced apoptosis by maintaining the expression level of H3K4me2. *Drug Deliv.* 25 1033–1043. 10.1080/10717544.2018.1461277 30799660 PMC6058728

[B42] Li D. Yan X. Hu Y. Liu Y. Guo R. Liao M. (2019). Two-photon image tracking of neural stem cells via iridium complexes encapsulated in polymeric nanospheres. *ACS Biomater. Sci. Eng.* 5 1561–1568. 10.1021/acsbiomaterials.8b01231 33405629

[B43] Li G. Chen K. You D. Xia M. Li W. Fan S. (2019). Laminin-coated electrospun regenerated silk fibroin mats promote neural progenitor cell proliferation, differentiation, and survival in vitro. *Front. Bioeng. Biotechnol.* 7:190. 10.3389/fbioe.2019.00190 31448271 PMC6691020

[B44] Li H. Edin F. Hayashi H. Gudjonsson O. Danckwardt-Lillieström N. Engqvist H. (2017). Guided growth of auditory neurons: bioactive particles towards gapless neural - electrode interface. *Biomaterials* 122 1–9. 10.1016/j.biomaterials.2016.12.020 28107660

[B45] Li J. Ding L. (2020). Effectiveness of steroid treatment for sudden sensorineural hearing loss: a meta-analysis of randomized controlled trials. *Ann. pharmacother.* 54 949–957. 10.1177/1060028020908067 32126823

[B46] Liu L. Chen Y. Qi J. Zhang Y. He Y. Ni W. (2016). Wnt activation protects against neomycin-induced hair cell damage in the mouse cochlea. *Cell Death Dis.* 7:e2136. 10.1038/cddis.2016.35 26962686 PMC4823936

[B47] Liu W. Xu L. Wang X. Zhang D. Sun G. Wang M. (2021). PRDX1 activates autophagy via the PTEN-AKT signaling pathway to protect against cisplatin-induced spiral ganglion neuron damage. *Autophagy* 17, 1–23. 10.1080/15548627.2021.1905466 33749526 PMC8726717

[B48] Liu W. Xu X. Fan Z. Sun G. Han Y. Zhang D. (2019). Wnt signaling activates TP53-induced glycolysis and apoptosis regulator and protects against cisplatin-induced spiral ganglion neuron damage in the mouse cochlea. *Antioxid. Redox Signal.* 30 1389–1410. 10.1089/ars.2017.7288 29587485

[B49] Liu Y. Chen Q. Xu Y. (2020). Research progress in refractory sudden hearing loss: steroid therapy. *J. Intern. Med. Res.* 48:300060519889426. 10.1177/0300060519889426 31939327 PMC7254608

[B50] Liu Y. Qi J. Chen X. Tang M. Chu C. Zhu W. (2019). Critical role of spectrin in hearing development and deafness. *Sci. Adv.* 5:eaav7803. 10.1126/sciadv.aav7803 31001589 PMC6469942

[B51] Liu Z. Tang M. Zhao J. Chai R. Kang J. (2018). Looking into the future: toward advanced 3D biomaterials for stem-cell-based regenerative medicine. *Adv. Mater.* 30:e1705388. 10.1002/adma.201705388 29450919

[B52] Lv J. Fu X. Li Y. Hong G. Li P. Lin J. (2021). Deletion of Kcnj16 in mice does not alter auditory function. *Front. Cell Dev. Biol.* 9:630361. 10.3389/fcell.2021.630361 33693002 PMC7937937

[B53] Mamo S. K. Nieman C. L. Lin F. R. (2016). Prevalence of untreated hearing loss by income among older adults in the United States. *J. Health Care Poor Under.* 27 1812–1818. 10.1353/hpu.2016.0164 27818440 PMC7307013

[B54] McCall A. A. Swan E. E. Borenstein J. T. Sewell W. F. Kujawa S. G. McKenna M. J. (2010). Drug delivery for treatment of inner ear disease: current state of knowledge. *Ear Hear.* 31 156–165. 10.1097/AUD.0b013e3181c351f2 19952751 PMC2836414

[B55] Mirian C. Ovesen T. (2020). Intratympanic vs systemic corticosteroids in first-line treatment of idiopathic sudden sensorineural hearing loss: a systematic review and meta-analysis. *JAMA Otolaryngol. Head Neck Surg.* 146 421–428. 10.1001/jamaoto.2020.0047 32163109 PMC7068668

[B56] Mokoena D. R. George B. P. Abrahamse H. (2019). Enhancing breast cancer treatment using a combination of cannabidiol and gold nanoparticles for photodynamic therapy. *Intern. J. Mol. Sci.* 20:4771. 10.3390/ijms20194771 31561450 PMC6801525

[B57] Murugadoss S. Vinković Vrček I. Pem B. Jagiello K. Judzinska B. Sosnowska A. (2021). A strategy towards the generation of testable adverse outcome pathways for nanomaterials. *Altex* 10.14573/altex.2102191 [Epub ahead of print]. 34008034

[B58] Nyberg S. Abbott N. J. Shi X. Steyger P. S. Dabdoub A. (2019). Delivery of therapeutics to the inner ear: the challenge of the blood-labyrinth barrier. *Sci. Transl. Med.* 11:eaao0935. 10.1126/scitranslmed.aao0935 30842313 PMC6488020

[B59] Pyykkö I. Zou J. Schrott-Fischer A. Glueckert R. Kinnunen P. (2016). An overview of nanoparticle based delivery for treatment of inner ear disorders. *Methods Mol. Biol.* 1427 363–415. 10.1007/978-1-4939-3615-1_21 27259938

[B60] Qi J. Liu Y. Chu C. Chen X. Zhu W. Shu Y. (2019). A cytoskeleton structure revealed by super-resolution fluorescence imaging in inner ear hair cells. *Cell Discov.* 5:12. 10.1038/s41421-018-0076-4 30792888 PMC6379372

[B61] Qi J. Zhang L. Tan F. Liu Y. Chu C. Zhu W. (2020). Espin distribution as revealed by super-resolution microscopy of stereocilia. *Am. J. Transl. Res.* 12 130–141. 32051742 PMC7013225

[B62] Rathnam C. Chueng S. D. Ying Y. M. Lee K. B. Kwan K. (2019). Developments in bio-inspired nanomaterials for therapeutic delivery to treat hearing loss. *Front. Cell. Neurosci.* 13:493. 10.3389/fncel.2019.00493 31780898 PMC6851168

[B63] Ray A. Mukundan A. Xie Z. Karamchand L. Wang X. Kopelman R. (2014). Highly stable polymer coated nano-clustered silver plates: a multimodal optical contrast agent for biomedical imaging. *Nanotechnology* 25:445104. 10.1088/0957-4484/25/44/445104 PMC4244271 25325364

[B64] Salazar-Alvarez G. Qin J. Sepelák V. Bergmann I. Vasilakaki M. Trohidou K. N. (2008). Cubic versus spherical magnetic nanoparticles: the role of surface anisotropy. *J. Am. Chem. Soc.* 130 13234–13239. 10.1021/ja0768744 18783216

[B65] Salt A. N. Hartsock J. Plontke S. LeBel C. Piu F. (2011). Distribution of dexamethasone and preservation of inner ear function following intratympanic delivery of a gel-based formulation. *Audiol. Neurootol.* 16 323–335. 10.1159/000322504 21178339 PMC3023000

[B66] Sarreal R. R. Bhatti P. (2020). Characterization and miniaturization of silver-nanoparticle microcoil via aerosol jet printing techniques for micromagnetic cochlear stimulation. *Sensors* 20:6087. 10.3390/s20216087 33114773 PMC7663185

[B67] Schuknecht H. F. (1957). Ablation therapy in the management of Menière’s disease. *Acta Otolaryngol. Suppl.* 132 1–42. 13457879

[B68] Shaikh S. Rehman F. U. Du T. Jiang H. Yin L. Wang X. (2018). Real-time multimodal bioimaging of cancer cells and exosomes through biosynthesized iridium and iron nanoclusters. *ACS Appl. Mater. Interf.* 10 26056–26063. 10.1021/acsami.8b08975 30011179

[B69] Shang L. Yu Y. Gao W. Wang Y. Qu L. Zhao Z. (2018). Bio-inspired anisotropic wettability surfaces from dynamic ferrofluid assembled templates. *Adv. Funct. Mater.* 28 148–155.

[B70] Shi X. (2016). Pathophysiology of the cochlear intrastrial fluid-blood barrier (review). *Hear. Res.* 338 52–63. 10.1016/j.heares.2016.01.010 26802581 PMC5322264

[B71] Stout A. Friedly J. Standaert C. J. (2019). Systemic absorption and side effects of locally injected glucocorticoids. *J. Injury Funct. Rehabil.* 11 409–419. 10.1002/pmrj.12042 30925034

[B72] Sun G. Liu W. Fan Z. Zhang D. Han Y. Xu L. (2016). The three-dimensional culture system with matrigel and neurotrophic factors preserves the structure and function of spiral ganglion neuron in vitro. *Neural Plastic.* 2016:4280407. 10.1155/2016/4280407 27057364 PMC4736769

[B73] Tan F. Chu C. Qi J. Li W. You D. Li K. (2019). AAV-ie enables safe and efficient gene transfer to inner ear cells. *Nat. Commun.* 10:3733. 10.1038/s41467-019-11687-8 31427575 PMC6700137

[B74] Tang M. Li J. He L. Guo R. Yan X. Li D. (2019). Transcriptomic profiling of neural stem cell differentiation on graphene substrates. *Colloids Surf. B Biointerf.* 182:110324. 10.1016/j.colsurfb.2019.06.054 31288132

[B75] Varela-Nieto I. Murillo-Cuesta S. Calvino M. Cediel R. Lassaletta L. (2020). Drug development for noise-induced hearing loss. *Expert Opin. Drug Discov.* 15 1457–1471. 10.1080/17460441.2020.1806232 32838572

[B76] Viola G. Chang J. Maltby T. Steckler F. Jomaa M. Sun J. (2020). Bioinspired multiresonant acoustic devices based on electrospun piezoelectric polymeric nanofibers. *ACS Appl. Mater. Interf.* 12 34643–34657. 10.1021/acsami.0c09238 32639712 PMC7460092

[B77] Wang T. Chai R. Kim G. S. Pham N. Jansson L. Nguyen D. H. (2015). Lgr5+ cells regenerate hair cells via proliferation and direct transdifferentiation in damaged neonatal mouse utricle. *Nat. Commun.* 6:6613. 10.1038/ncomms7613 25849379 PMC4391285

[B78] Wang X. Dellamary L. Fernandez R. Harrop A. Keithley E. M. Harris J. P. (2009). Dose-dependent sustained release of dexamethasone in inner ear cochlear fluids using a novel local delivery approach. *Audiol. Neurootol.* 14 393–401. 10.1159/000241896 19923809

[B79] Waqas M. Sun S. Xuan C. Fang Q. Zhang X. Islam I. U. (2017). Bone morphogenetic protein 4 promotes the survival and preserves the structure of flow-sorted Bhlhb5+ cochlear spiral ganglion neurons in vitro. *Sci. Rep.* 7:3506. 10.1038/s41598-017-03810-w 28615657 PMC5471210

[B80] Wei H. Chen Z. Hu Y. Cao W. Ma X. Zhang C. (2021). Topographically conductive butterfly wing substrates for directed spiral ganglion neuron growth. *Small* 2021:e2102062. 10.1002/smll.202102062 34411420

[B81] Xia L. Shang Y. Chen X. Li H. Xu X. Liu W. (2020). Oriented neural spheroid formation and differentiation of neural stem cells guided by anisotropic inverse opals. *Front. Bioeng. Biotechnol.* 8:848. 10.3389/fbioe.2020.00848 32850719 PMC7411081

[B82] Xia L. Zhu W. Wang Y. He S. Chai R. (2019). Regulation of neural stem cell proliferation and differentiation by graphene-based biomaterials. *Neural Plastic.* 2019:3608386. 10.1155/2019/3608386 31737061 PMC6817925

[B83] Yan W. Liu W. Qi J. Fang Q. Fan Z. Sun G. (2018). A three-dimensional culture system with matrigel promotes purified spiral ganglion neuron survival and function in vitro. *Mol. Neurobiol.* 55 2070–2084. 10.1007/s12035-017-0471-0 28283883

[B84] Yang Y. Gao B. Hu Y. Wei H. Zhang C. Chai R. (2021). Ordered inverse-opal scaffold based on bionic transpiration to create a biomimetic spine. *Nanoscale* 13 8614–8622. 10.1039/d1nr00731a 33929471

[B85] Yang Y. Zhang Y. Chai R. Gu Z. (2018). Designs of biomaterials and microenvironments for neuroengineering. *Neural Plastic.* 2018:1021969. 10.1155/2018/1021969 30627148 PMC6304813

[B86] Yang Y. Zhang Y. Chai R. Gu Z. A. (2020). Polydopamine-functionalized carbon microfibrous scaffold accelerates the development of neural stem cells. *Front. Bioengin. Biotechnol.* 8:616. 10.3389/fbioe.2020.00616 32714901 PMC7344254

[B87] Yoon J. Y. Yang K. J. Kim D. E. Lee K. Y. Park S. N. Kim D. K. (2015). Intratympanic delivery of oligoarginine-conjugated nanoparticles as a gene (or drug) carrier to the inner ear. *Biomaterials* 73 243–253. 10.1016/j.biomaterials.2015.09.025 26414408

[B88] Yuan T. F. Dong Y. Zhang L. Qi J. Yao C. Wang Y. (2021). Neuromodulation-based stem cell therapy in brain repair: recent advances and future perspectives. *Neurosci. Bull.* 37 735–745. 10.1007/s12264-021-00667-y 33871821 PMC8099989

[B89] Zahnert T. (2011). The differential diagnosis of hearing loss. *Deutsches Arzteblatt Intern.* 108 433–443. 10.3238/arztebl.2011.0433 21776317 PMC3139416

[B90] Zha Y. Chai R. Song Q. Chen L. Wang X. Cheng G. (2016). Characterization and toxicological effects of three-dimensional graphene foams in rats in vivo. *J. Nanopartic. Res.* 18:122.

[B91] Zhang S. Liu D. Dong Y. Zhang Z. Zhang Y. Zhou H. (2019). Frizzled-9+ supporting cells are progenitors for the generation of hair cells in the postnatal mouse cochlea. *Front. Mol. Neurosci.* 12:184. 10.3389/fnmol.2019.00184 31427926 PMC6689982

[B92] Zhang Y. Li W. He Z. Wang Y. Shao B. Cheng C. (2019). Pre-treatment with fasudil prevents neomycin-induced hair cell damage by reducing the accumulation of reactive oxygen species. *Front. Mol. Neurosci.* 12:264. 10.3389/fnmol.2019.00264 31780893 PMC6851027

[B93] Zhang S. Zhang Y. Dong Y. Guo L. Zhang Z. Shao B. (2020b). Knockdown of Foxg1 in supporting cells increases the trans-differentiation of supporting cells into hair cells in the neonatal mouse cochlea. *Cell. Mol. Life Sci.* 77 1401–1419. 10.1007/s00018-019-03291-2 31485717 PMC7113235

[B94] Zhang S. Qiang R. Dong Y. Zhang Y. Chen Y. Zhou H. (2020a). Hair cell regeneration from inner ear progenitors in the mammalian cochlea. *Am. J. Stem Cells* 9 25–35. 32699655 PMC7364385

[B95] Zhang Y. Zhang S. Zhang Z. Dong Y. Ma X. Qiang R. (2020c). Knockdown of Foxg1 in Sox9+ supporting cells increases the trans-differentiation of supporting cells into hair cells in the neonatal mouse utricle. *Aging* 12 19834–19851. 10.18632/aging.104009 33099273 PMC7655167

[B96] Zhang Y. Z. Wang Y. Jiang Q. El-Demellawi J. K. Kim H. Alshareef H. N. (2020d). MXene printing and patterned coating for device applications. *Adv. Mater.* 32:e1908486. 10.1002/adma.201908486 32239560

[B97] Zhao C. Chen G. Wang H. Zhao Y. Chai R. (2021). Bio-inspired intestinal scavenger from microfluidic electrospray for detoxifying lipopolysaccharide. *Bioactive Mater.* 6 1653–1662. 10.1016/j.bioactmat.2020.11.017 33313445 PMC7701841

[B98] Zhao E. E. Dornhoffer J. R. Loftus C. Nguyen S. A. Meyer T. A. Dubno J. R. (2020). Association of patient-related factors with adult cochlear implant speech recognition outcomes: a meta-analysis. *JAMA Otolaryngol. Head Neck Surg.* 146 613–620. 10.1001/jamaoto.2020.0662 32407461 PMC7226297

[B99] Zhao J. Tang M. Cao J. Ye D. Guo X. Xi J. (2019). Structurally tunable reduced graphene oxide substrate maintains mouse embryonic stem cell pluripotency. *Adv. Sci.* 6:1802136. 10.1002/advs.201802136 31380157 PMC6662269

[B100] Zhong Z. Fu X. Li H. Chen J. Wang M. Gao S. (2020). Citicoline protects auditory hair cells against neomycin-induced damage. *Front. Cell Dev. Biol.* 8:712. 10.3389/fcell.2020.00712 32984303 PMC7487320

[B101] Zhou H. Ma X. Liu Y. Dong L. Luo Y. Zhu G. (2015). Linear polyethylenimine-plasmid DNA nanoparticles are ototoxic to the cultured sensory epithelium of neonatal mice. *Mol. Med. Rep.* 11 4381–4388. 10.3892/mmr.2015.3306 25652676

[B102] Zhou H. Qian X. Xu N. Zhang S. Zhu G. Zhang Y. (2020). Disruption of Atg7-dependent autophagy causes electromotility disturbances, outer hair cell loss, and deafness in mice. *Cell Death Dis.* 11:913. 10.1038/s41419-020-03110-8 33099575 PMC7585579

[B103] Zhu C. Cheng C. Wang Y. Muhammad W. Liu S. Zhu W. (2018). Loss of ARHGEF6 causes hair cell stereocilia deficits and hearing loss in mice. *Front. Mol. Neurosci.* 11:362. 10.3389/fnmol.2018.00362 30333726 PMC6176010

[B104] Zou J. Hannula M. Misra S. Feng H. Labrador R. H. Aula A. S. (2015). Micro CT visualization of silver nanoparticles in the middle and inner ear of rat and transportation pathway after transtympanic injection. *J. Nanobiotechnol.* 13:5. 10.1186/s12951-015-0065-9 25622551 PMC4312601

[B105] Zou J. Ostrovsky S. Israel L. L. Feng H. Kettunen M. I. Lellouche J. M. (2017b). Efficient penetration of ceric ammonium nitrate oxidant-stabilized gamma-maghemite nanoparticles through the oval and round windows into the rat inner ear as demonstrated by MRI. *J. Biomed. Mater. Res. Part B Appl. Biomater.* 105 1883–1891. 10.1002/jbm.b.33719 27239906

[B106] Zou J. Feng H. Sood R. Kinnunen P. K. J. Pyykko I. (2017a). Biocompatibility of liposome nanocarriers in the rat inner ear after intratympanic administration. *Nanoscale Res. Lett.* 12:372. 10.1186/s11671-017-2142-5 28549377 PMC5445035

